# Could a Change in Diet Revitalize Children Who Suffer from Unresolved Fatigue?

**DOI:** 10.3390/nu7031965

**Published:** 2015-03-13

**Authors:** Tessa Gerjanne Steenbruggen, Sietske Johanna Hoekstra, Ellen José van der Gaag

**Affiliations:** 1 University Medical Centre Groningen, Hanzeplein 1, 9700 RB Groningen, The Netherlands; E-Mails: t.g.steenbruggen@gmail.com (T.G.S.); hoekstrasj@gmail.com (S.J.H.); 2 Ziekenhuisgroep Twente, Hengelo, Geerdinksweg 141, 7555 DL Hengelo, The Netherlands

**Keywords:** children, micronutrients, fatty acids, nutrient intake, fatigue, vegetables

## Abstract

Many children deal with fatigue for which no proper treatment can be given. A possible explanation for their fatigue is a micro deficiency of minerals and vitamins. In this non-randomized controlled trial, we clinically evaluated symptoms of fatigue in children for whom a nutrient-rich diet was advised. A group of 98 children (2–18 years old) with unexplained symptoms of fatigue was examined. The dietary modifications consisted of green vegetables, beef, whole milk and full-fat butter. Children in the intervention group were asked to follow the diet for three months, whereas the control-group followed their normal diet. The primary outcome was symptoms of fatigue, as determined by a PedsQL Multidimensional Fatigue Scale, and secondary outcomes were compliance with the diet and BMI. Children, who followed the diet showed a significant decrease in the need to sleep (CI 0.83; 14.86, *p* = 0.03). They slept better through the night and took fewer naps. When analyzing components of the advised diet separately, a significant larger decrease in cognitive fatigue symptoms was seen for eating green vegetables according to the diet guidelines (CI 2.27; 30.63, *p* = 0.024). Furthermore, a lower need to sleep was seen when whole milk was consumed almost daily (CI 0.02; 14.62, *p* = 0.049). Our study showed that nutritional advice is an elegant, and effective method for decreasing some symptoms of medically unresolved fatigue in children.

## 1. Introduction

Unresolved fatigue affects many children: 1.4% of children under 12% and 2.6% of children between 12 and 18 in the Netherlands experience fatigue, as reported by general practitioners [1]. Self-reporting of fatigue is even higher at 23%–26% in children under 12 and up to 34%–46% in teenagers [1,2,3]. Fatigue in children can cause many problems. They experience cognitive problems such as decreased concentration and school absenteeism, often resulting in less or a lower level of education [1,4,5]. Children who suffer from fatigue are less resilient, have lower immune resistance [6], and experience sleep disturbances [7]. Although fatigue is hard to define, it is often under-diagnosed by both general practitioners and pediatricians. 

Fatigue can have many different causes; however, without a medical cause for the fatigue, pediatricians or general practitioners cannot start a therapy. Once infections, anemia, chronic disease, and psychosocial imbalance are ruled out, there is still a group of patients without a clear cause and children for whom adequate medical therapy does not help. A possible cause for children with unresolved fatigue is a micro deficiency of vitamins and minerals, which can be difficult to detect [8,9]. Iron deficiency without anemia has been shown to cause fatigue and impair cognitive function in children and adolescent girls [10,11]. Individual micronutrients including iron [10,11], magnesium [12,13], vitamin B [14], vitamin C [15], vitamin E [16,17], and fatty acids [18,19] have been shown to have an effect on fatigue. Clinical studies show that approximately 20% of children under eight do not eat enough fruit and vegetables and do not reach the minimum level of dairy intake (according to European guidelines: EFSA) [20,21,22].

A further problem that can develop is chronic fatigue syndrome (CFS). More research has been done about the effects of nutrients on clinical symptoms in patients with CFS. A diet with enhanced vitamins and minerals in combination with psychological training significantly improves symptomatology and fatigue in CFS-patients [23]. However, the patients in the CFS study were individually selected for the combined intervention and without a control group it is difficult to prove statistical significance and apply their results in the clinic. 

Finally, previous studies in our hospital have shown that a nutrient-rich diet had a positive effect on recurrent upper airway infections and on subclinical hypothyroidism in children [6,24]. The components were chosen based on their high vitamin and mineral content [25]. The advised diet contains more vitamins A, B2, B12, C, D and E, iron, folic acid, zinc, calcium, phosphorus, Beta-carotene, and (alpha)-linoleic acid than a regular diet (Table 1 and Table S1 in supplementary materials) [25].

This study was conducted as a prospective, non-randomized controlled trial, which focuses on a dietary advice as an intervention to reduce fatigue in children. 

## 2. Experimental Section

### 2.1. Study Population

Children (2–18 years old) who were referred by a general practitioner to a pediatrician with (subjective) complaints of fatigue between September 2011 and February 2012 were included in this study. Their fatigue could not be explained by interview, physical examination, or laboratory blood tests (Hb, Ht, MCV, MCH, MCHC, RDW, thrombocytes, leukocytes (including differentiation), TSH, FT4, 25-OH-vitamin-D3, IgE and allergy screening). In cases of a possible medical condition, a treatment was started and they were no longer appropriate for this study. 

Children with fatigue due to an untreated underlying somatic or mental disease or with low pediatric quality of life inventory (PedsQL) scores (>98.0) were excluded. 

When children were treated for their underlying somatic disease, and there was no improvement of the fatigue with appropriate treatment (e.g., supplementation of vitamin D or the use of anti-histamine), they could also be included. 

The control group consisted of children that visited the same outpatient clinic for fatigue symptoms, in the period from March 2011 until August 2011 (six months before the treatment group). The same inclusion and exclusion criteria were used for both the control and the intervention group. 

**Table 1:** Comparison regular diet and advised diet based on regular dietary intake for a child 7 years of age.

Dependent variable	Regular Diet	Advised Diet
Vitamin A (μg)	169.00	198.80
Vitamin C (mg)	38.25	47.75
Vitamin D (μg)	1.57	0.51
Vitamin E (mg)	2.79	12.56
Vitamin B2 (mg)	2.39	2.30
Vitamin B12 (μg)	4.82	5.29
Iron (mg)	4.37	5.42
Zinc (mg)	5.48	11.24
Folic Acid (μg)	192.51	337.96
Phosphor (mg)	1745.80	1710.30
Calcium (mg)	1485.80	1327.30
Beta-caroten (μg)	5122.90	6035.90
(alpha)-linoleic acid (%)	40.62	44.74

The portions are based on a normal day menu according to the recommended daily intake for a child, 7 years of age, from the “Voedingscentrum”, the Netherlands.

### 2.2. Ethics

All children and their parents received oral information about the study and were asked to give their informed consent. The central committee on research involving human subjects judged that dietary advice that includes regular food is not a major lifestyle change, and therefore an evaluation by an ethics committee was not necessary for this study. The control group was treated with standard care and followed up as usual as no extra interventions were necessary. 

### 2.3. Intervention

Both groups received standard care. Standard care includes advice to keep an active lifestyle and to not spend too much time in bed. Furthermore a pediatrician followed up with regular checkups for all of the children. Children in the intervention group together with their parents received dietary advice from a pediatrician, which was comprised of four key components: green vegetables, beef, whole milk, and full-fat butter. Green vegetables should be eaten 5 times per week, in appropriate portions for the child’s age (e.g., 2–3 tablespoons per day for toddlers). Beef should be eaten 3 times per week (e.g., 1–2 tablespoons per day for toddlers). Furthermore, one cup (200 mL) of whole milk per day and full-fat butter on bread was recommended. All parts of the diet were weighted equally, with a total possible score of 100% (e.g., vegetables 4 times per week is 80% compliance for the vegetable part, and 20% of the total compliance score). For children under 12 years of age, their parents were asked to cook together with the children and keep a diary to measure compliance. The pediatrician checked up monthly to control compliance with a specific questionnaire. The pediatrician (E. van der Gaag) designed a questionnaire, which has been used in earlier studies [6,24].

The intervention was started one month after the screening and laboratory blood tests were done. The diet was followed over the course of at least 3 months, in which a pediatrician saw the children twice and checked up with them by phone monthly. After three months all children were evaluated with the PedsQL questionnaire.

### 2.4. Measurements

The PedsQL multidimensional fatigue scale (MAPI Research Trust), designed by Varni and colleagues, was used to evaluate fatigue before starting the diet and three months after the advice was given [26]. The PedsQL scale is a validated method to measure fatigue in children [26,27]. Fatigue was measured in three different ways: general fatigue, cognitive fatigue, and the need to sleep or rest, with 6 questions for each type. General fatigue measures the level of energy for normal daily activities (e.g., “I feel too tired to do the things I like.”), cognitive fatigue measures the impact of fatigue on the level of concentration (e.g., “It is hard for me to keep my attention on things.”), and the need to sleep measures how much time a child spends in bed or needs to rest (e.g., “I rest a lot.”). Each question was answered on a five-point scale (0 = never, 1 = almost never, 2 = sometimes, 3 = almost always and 4 = always). Each score from the questionnaire was transformed to a 0–100 scale, where a higher score reflects a lower impact of symptoms. Results were excluded when less than half of the questionnaire was filled out. 

To check the compliance to the diet we used a simple questionnaire, which has been used in earlier studies with the same advised diet [6,24]. 

Furthermore, weight and height were measured with the same measurement devices at the start and after 3 months so that body mass index (BMI) changes could be evaluated. 

### 2.5. Statistics

Continuous variables are displayed as means (standard deviation (SD)) and categorical variables are displayed as numbers with corresponding percentages. 

We used descriptive statistics to explore our results. A chi-square test was used to compare baseline categorical characteristics of the intervention and control groups, whereas an independent sample *t*-test was used for continuous variables, such as BMI. Afterwards, compliance with the diet between both groups was analyzed with an independent sample *t*-test. Furthermore, the effect of the diet on fatigue was analyzed with an analysis of covariance (ANCOVA). The data were first analyzed on an “intention to treat” basis, comparing the intervention group with the control group. Finally, a “per protocol” analysis was done, in which patients that did not follow the diet were removed, to evaluate the maximum possible effect. In the “per protocol” analysis children following the diet with a great than 75% score were included in the study. To correct for possible confounders, a multiple linear regression analysis was done for the overall diet and for the separate components. 

To analyze the correlation between the compliance to the advised diet and change in score on the PedsQL a Spearman rank correlation analysis was performed. 

For all analyses a *p*-value of 0.05 was used to define a significant difference. For the selection of confounders a *p*-value of 0.15 was used. 

All statistical analyses were done with the program SPSS version 22.0. 

## 3. Results

### 3.1. Study Population

109 children were recruited, of which 98 were analyzed: 48 children were included between March 2011 and September 2011 and served as controls, 50 children were included between September 2011 and March 2012 and were put in the intervention group. Eleven children were excluded, as they did not meet the inclusion criteria: 3 children had an explanation for their fatigue, 2 children were too young, 3 children scored too high on the PedsQL questionnaire (PedsQL > 98; which means their subjective feeling of fatigue could not be found in objective questionnaires), and 3 children from the control group were lost to follow-up. In the “per protocol” analysis 88 children were included: 48 in the control group and 40 in the intervention group. 

Demographic characteristics of the study population are shown in Table 2. In the intervention group 27 children (54%) had comorbidity on top of their fatigue, compared to 26 children (54%) in the control group. These comorbidities included asthma or upper respiratory tract infection (URTI) or subclinical hypothyroidism and were similar for both groups (see Table S2 for more details). 

### 3.2. Compliance of Intervention Group

At the start of the study, children from the control group already followed the dietary advice for 29%, whereas children in the intervention group did also for 29%. The compliance increased spontaneously to 32% in the control group and to 85% in the intervention group. This difference in compliance was statistically significant (95% CI −0.58; −0.44, *p* = 0.00). 

### 3.3. Clinical Outcomes

A decline in symptoms was seen across all components of the total PedsQL score in both groups, but with a stronger decline in the intervention group (see Table 3). In the intervention group the mean score for the need to sleep or rest increased with 12.0 points, compared to an increase of 4.4 points in the control group (95% CI 0.83; 14.86, *p* = 0.03). This means, the children showed a decreased need to sleep or take naps and spent less time in bed during the day. General fatigue improved with 9.5 points compared to 6.4 points in the control group and cognitive fatigue with 8.1 points and 2.9 for the intervention and control group respectively. However, the results were not significantly different (general fatigue: 95% CI −3.35; 11.09, *p* = 0.29; cognitive fatigue: 95% CI −6.58; 11.90, *p* = 0.57, respectively). 

**Table 2:** Demographic characteristics baseline.

*N* = 98	Intervention Group (*n* = 50)	Control Group (*n* = 48)	*p*-value
Sex (M/F)	24/26	21/27	0.69
Age mean (SD) (years)	7.44 (5.0)	7.0 (4.8)	0.66
FU time mean (SD) (days)	137.1 (67.0)	141.9 (72.8)	0.73
General fatigue *	49.6 (19.0)	47.9 (24.2)	0.70
Cognitive fatigue *	55.3 (22.1)	60.0 (24.6)	0.33
Sleep/need to rest *	57.1 (20.4)	55.8 (18.7)	0.74
Comorbid diagnosis (%)	27 (54) ^§^	26 (54) ^§^	0.41
Medication (%)	25 (50) ^§^	28 (58) ^§^	0.99
Compliance to the total diet (%)	29.8	29.3	0.91
Compliance to green vegetables (%)	47.1	56.5	0.26
Compliance to beef (%)	40.6	54.9	0.13
Compliance to whole milk (%)	17.0	8.6	0.24
Compliance to full fat butter (%)	12.5	2.5	0.09

* Expressed as points on PedsQL scale (range 0–100); ^§^ Specified in Table S2 in supplementary materials.

**Table 3:** Mean scores (range) after following the diet for three months and change (SD) in symptoms of fatigue measured by pediatric quality of life (PedsQL) scores.

Dependent variable	Intervention Group (*N* = 40)	Control Group (*N* = 48)
Second score (range) PedsQL		
General fatigue	59.1 (16.7–91.7)	54.4 (4.2–100.0)
Cognitive fatigue	66.7 (0.0–100.0)	63.8 (16.7–100.0)
Need to sleep	66.5 (29.2–95.8)	60.1 (16.7–100.0)
Change (SD) in score PedsQL		
General fatigue	9.5 (17.5)	6.4 (19.2)
Cognitive fatigue	8.1 (21.4)	2.9 (18.4)
Need to sleep	12.0 * (17.8)	4.4 * (16.3)

PedsQL scale: 0–100; Change shown as second score minus baseline score; * *p* < 0.05 for difference between intervention and control group.

Analyzing the correlation between the rate of compliance and the effect of the advised diet showed similar results. A significant correlation was only seen for the variable “need to sleep” (data shown in Figure S1 in supplementary materials).

The difference in fatigue symptoms was also studied for the individual components of the diet, while taking confounders into account (e.g., first measurement of fatigue symptoms and a change in BMI).

Green vegetables were shown to have a significant effect on cognitive fatigue (difference: 16.45, 95% CI 2.27; 30.63, *p* = 0.024). Green vegetables also showed a trend to improve general fatigue and reduced the need to sleep but not to significant difference. Furthermore, whole milk had a significant impact on the need to sleep (difference: 7.32, 95% CI 0.02; 14.62, *p* = 0.049) (Table 4). 

**Table 4:** Analysis of Covariance of the effect of components of the diet on symptoms of fatigue.

Dependent variable	Green Vegetables		Beef		Whole Milk		Full-Fat Butter	
*N* = 88	B *	95% CI	B *	95% CI	B *	95% CI	B *	95% CI
General fatigue	8.88	−1.23; 18.99	1.32	−10.12; 12.75	3.62	−6.26; 13.50	−2.55	−12.07; 6.97
Cognitive fatigue	16.45	2.27; 30.63	3.83	−7.44; 15.10	−1.70	−11.26; 7.86	−1.20	−10.47; 8.08
Need to sleep	8.12	−2.74; 18.98	7.56	−0.94; 16.06	7.32	0.02; 14.62	2.63	−4.69; 9.96

All analyses were corrected for the baseline values of the fatigue symptoms as well as for the change in BMI between the baseline and follow-up measurement. * B represents the difference between the intervention and control group. A positive difference is in favor of the intervention group.

### 3.4. Impact on BMI

For 35 children a change in BMI was measured (17 in the control group, 18 in the intervention group. Generally, full-fat products like whole milk and butter are expected to lead to weight gain. However, measurements before and after the diet showed a change in BMI in the control group of −0.39 and in the intervention group of 0.06 (95% CI −0.147; 1.03, *p* = 0.139).

## 4. Discussion

### 4.1. Benefits of a Nutrient-Rich Diet

This non-randomized controlled study showed a decline in clinical complaints of fatigue in children following the advised diet, especially in the need to rest and sleep. Patients took fewer naps and spent less time in bed during the day when following the advised diet. The observed positive effect on fatigue can be explained by an increased intake of minerals, vitamins and fatty acids [18,19,28,29]. Four further factors can explain these results, including a beneficial effect of combining nutrients, effects of anti-oxidants, better sleep behavior due to the high concentration of melatonin in milk [30], and an improvement of the immune function.

Vitamins and minerals have been shown to mutually benefit their absorption when consumed simultaneously. The intake of green vegetables appeared to have the strongest impact on fatigue, especially for cognitive fatigue. This could be because vitamins A and C in green vegetables improve the absorption of other minerals such as zinc [31,32], magnesium [33] and iron [34]. Vitamin A can even reduce iron-dependent anemia [34,35]. Previous studies focused on individual nutritional supplements rather than on diets, which do not fully take into account this mutually beneficial effect. On top of this, it has been shown that pure nutrients are absorbed better than artificial nutrients [33]. For example, a study with an artificial polynutrient supplement (multivitamin pill) did not result in a significant difference in the feeling of fatigue [36]. A stronger impact on fatigue could therefore be explained by the pure nutrients in the advised diet *versus* artificial supplements.

Beside vitamins and minerals, green vegetables and melatonin in whole milk contain many anti-oxidants, which can reduce oxidative stress in the human body [30,37,38], which is associated with fatigue in children [16,39]. A reduction of oxidative stress by an increased consumption of green vegetables and whole milk could partly explain the decrease in fatigue. 

Melatonin has more beneficial properties on fatigue, especially on sleep behavior. Whole milk is known to contain high melatonin levels [40], which has been shown to be effective in improving sleep onset, maintenance of sleep, and prolongation of sleep [30,40]. Our study demonstrated that whole milk is significantly associated with a decreased need to sleep or rest, and that there was a stronger decline in fatigue in children who drank more whole milk than in children who drank less whole milk. As most children did not drink whole milk before the start of the diet, an increased supply of melatonin may have improved their sleeping behavior and feeling of wellbeing as well.Surprisingly, the effect of drinking whole milk was not related to the time it was consumed, as the result was also found when it was consumed in the morning or afternoon. We hypothesize that an increased ingestion of melatonin stimulates the body’s own production of melatonin at night.

Additionally, the components of the advised diet can improve the immune function in children. Earlier studies in our hospital showed that this diet has a beneficial effect on recurrent upper respiratory tract infections [6]. This is supported in the literature by an improvement of the immune functions by vitamins and minerals, which are present in high concentrations in the advised diet [41,42]. Even without recurrent infections, improvement of the immune system can be reflected in the general wellbeing, and an increase in energy due to less pro-inflammatory cytokines [43].

Finally, no significant change in BMI was measured in our study. We evaluated the effect of the advised diet on the BMI as full fat dairy products are generally not advised for children [44]. However, beneficial effects of full fat dairy products on asthma [45] and metabolic syndrome in children have been observed before, without an increase in BMI [29,46,47,48,49,50]. We therefore encourage the advised normal portions of full fat dairy products.

As the prevalence of fatigue in children is high, therapeutic options are limited, and there is little research on the impact of nutrition, this study is highly clinically relevant. We saw a clear clinical improvement in fatigue, which was also noticed by the children’s parents. Children seemed healthier and more resilient, and their parents observed less need to sleep or rest and more energy for daily activities. Moreover, the diet is an elegant, safe, low-cost, and easy treatment for fatigue and can be combined with therapy for other medical conditions.

### 4.2. Limitations and Future Perspectives

Although the results of this study are highly relevant, there are several limitations. Firstly, the study groups were small and not randomized because this was the first trial to investigate the effect between a nutrient rich diet and fatigue. Furthermore, the intervention was not performed in a double-blind fashion. Although the PedsQL is a validated method for objectifying fatigue [26,27], it remains a subjective measure. The group of children studied consists of children who are simply fatigued and children with comorbidities and use of medication. The percentage of children with comorbidities and medication-usage was the same in both groups but could still influence the effect of the advised diet. A sensitivity analysis showed that the difference in effect observed in PedsQL between the children with and without the diet was even greater. While the children with the advised diet showed similar results, children in the control group showed minimal improvement (data not shown). This indicates that also in children without comorbidities and use of medication, the observed effect of the advised diet was present and that this effect might even be underestimated. However, the group in which the sensitivity analysis was performed was very small and therefore statically relevant conclusions cannot be drawn from this analysis.

Second, laboratory markers were not analyzed to show the effect or the mechanism of the diet on micronutrient deficiencies, as this study focused on the clinical effect. In our study group, laboratory values could not be used, since micro deficiencies are not measurable and patients were excluded once abnormalities were found in the initial laboratory research. It would be interesting to measure laboratory markers, to clarify the mechanism through which the effect of the diet has occurred. A future study could focus on measuring laboratory markers and further quantifying the mechanism of the diet.

A third restraint is the possibility that the period in which children were included in the control group and intervention group affects the results. The control group was studied in the summer (March to August), whereas the intervention group was studied in the winter (September to February). Children visit a general practitioner or pediatrician more often during the winter months than in summer for all kinds of illnesses [51,52], and a decrease in vitality is reported during winter in adolescent girls [53]. This suggests that within both the intervention group and the control group, children could be on the up- or the downswing of a seasonal cycle. For example, a child that starts the diet in February may feel less tired in May regardless of the diet. Overall, it would be interesting (and fair) to evaluate the effect of the advised diet in a simultaneous period and potentially with a longer follow-up. 

Fourth, the different components of the diet were valued at the same level. However, this does not reflect the consumption of each of the components, and potentially does not reflect the relative benefits of each of the components. Furthermore, the questionnaire used to measure compliance is not validated.

Finally, parents and children only received advice and an explanation about why the components in the diet are important, rather than a prescription to follow the diet. We used a non-validated questionnaire to check the compliance to the advised diet. However, this questionnaire appeared adequate in other studies in which we used it to check the compliance to the same advised diet [6,24]. The “per protocol” analysis showed that the effect of the diet was stronger when it was followed more strictly (“intention to treat” analysis is not shown). Furthermore, a significant relationship between the degree of compliance and the need to sleep was found, which mirrors the results for the total sample. 

In a new study, more rigorous check-ups with parents and children to ensure it is followed could increase the effectiveness of the diet.

## 5. Conclusions

Many children deal with the impact of fatigue for which an explanation is lacking and good treatment is unavailable. A nutrient-rich diet is an elegant, effective, and low-cost method to demonstrate clinical improvement in children with these unexplained symptoms of fatigue. The micronutrients in green vegetables and in whole milk have an especially strong effect on fatigue. More research in a larger, randomized trial with more supervision on the dietary intake could further validate the results shown here.

## Supplementary Files

Supplementary File 1
