# Supplementary material for: Could a Change in Diet Revitalize Children Who Suffer from Unresolved Fatigue?
Source: Nutrients. 2015 Mar 13;7(3):1965–77. doi: 10.3390/nu7031965 (PMC4377893; doi:10.3390/nu7031965)
Supplement: Supplementary File 1 [file nutrients-07-01965-s001.docx]

**Supplementary Materials**

**Table S1.** Nutrients per 100 gram.

| **Dependent variable** | **Green Vegetables** | | **Beef** | **Full-Fat Butter** | **Whole Milk** |
| --- | --- | --- | --- | --- | --- |
|  | **Salad** | **Spinach** |  |  |  |
| Vitamins | | | | | |
| Vit A1 (ug) | 0.00 | 0.00 | 62.00 | 829.00 | 34.00 |
| Vit B1 (mg) | 0.04 | 0.05 | 0.07 | 0.01 | 0.03 |
| Vit B2 (mg) | 0.05 | 0.15 | 0.16 | 0.03 | 0.18 |
| Vit B12 (ug) | 0.00 | 0.00 | 2.31 | 0.30 | 0.39 |
| Vit B6 total (mg) | 0.06 | 0.08 | 0.17 | 0.00 | 0.034 |
| Folate (ug) | 24.50 | 124.50 | 12.80 | Trace | 6.50 |
| Vit C (mg) | 2.00 | 7.00 | 7.00 | 0.00 | 0.00 |
| Vit D total (mg) | 0.00 | 0.00 | 0.50 | 1.20 | 0.00 |
| Vit E total (mg) | 0.20 | 3.50 | 2.40 | 1.90 | 0.10 |
| Essential fatty acids | | | | | |
| Linoleic acid (mg) | - | - | - | 1.30 | 1.70 |
| α-Linoleic acid (mg) | - | - | - | 0.60 | 1.10 |
| Minerals | | | | | |
| Sodium (mg) | 14.00 | 15.00 | 440.00 | - | 43.00 |
| Potassium (mg) | 300.00 | 711.00 | 424.00 | - | 165.00 |
| Calcium (mg) | 51.00 | 84.00 | 19.00 | - | 102.00 |
| Phosphor (mg) | 35.00 | 71.00 | 218.00 | - | 102.00 |
| Magnesium (mg) | 14.00 | 77.00 | 27.00 | - | 12.00 |
| Iron total (mg) | 0.80 | 2.40 | 2.80 | - | 0.00 |
| Iron haem (mg) | 0.00 | 0.00 | 1.60 | - | 0.00 |
| Iron non haem (mg) | 0.80 | 2.40 | 1.20 | - | 0.00 |
| Copper (mg) | 0.04 | 0.08 | 0.10 | - | 0.00 |
| Selenium (ug) | 0.00 | 0.00 | 11.00 | - | 1.00 |
| Zinc (mg) | 0.33 | 1.20 | 6.27 | - | 0.46 |
| Iodine (ug) | 1.90 | 2.00 | 5.20 | - | 7.00 |

**Table S2.** Specification of comorbid diagnosis and medication.

| **Dependent variable** | **Intervention Group (*n* = 50)** | **Control Group (*n* = 48)** |
| --- | --- | --- |
| Comorbid diagnosis | | |
| Asthma/URTI | 12 | 9 |
| Subclinical hypothyroidism | 5 | 6 |
| Hyperthyroidism | 0 | 1 |
| Concentration disorder | 3 | 3 |
| Hypovitaminosis D | 3 | 2 |
| Anemia | 1 | 2 |
| Thalassemia | 0 | 1 |
| Obstipation | 1 |  |
| Coeliac disease | 2 | 1 |
| Sleep apnea | 2 | 1 |
| Migraine | 1 | 0 |
| IgA deficiency | 1 | 0 |

**Table S2.** *Cont.*

| **Dependent variable** | **Intervention Group (*n* = 50)** | **Control Group (*n* = 48)** |
| --- | --- | --- |
| Selective immunoglobulin  synthesis defect | 0 | 1 |
| Diabetes mellitus type 1 | 1 | 0 |
| Down syndrome | 1 | 0 |
| Epilepsy | 0 | 2 |
| Recidivate tachycardia | 0 | 1 |
| Hearing loss | 0 | 2 |
| Medication | | |
| Vitamin D | 14 | 9 |
| Inhalation sympathicomimetica | 9 | 3 |
| Inhalation corticosteroids | 3 | 3 |
| Inhalation antihistamine | 4 | 1 |
| Polyethylene glycol electrolyte solution | 3 | 1 |
| Clarithromycin | 1 | 0 |
| Co-trimoxazole | 1 | 0 |
| Amoxicillin | 0 | 1 |
| Ferrofumarate tablets | 1 | 6 |
| Thyrax | 1 | 3 |
| Insulin pomp | 1 | 0 |
| Levonorgestrel/-ethinylestradiol | 1 | 1 |
| Ritalin. | 0 | 1 |
| Depakine | 0 | 1 |
| Melatonin | 0 | 2 |


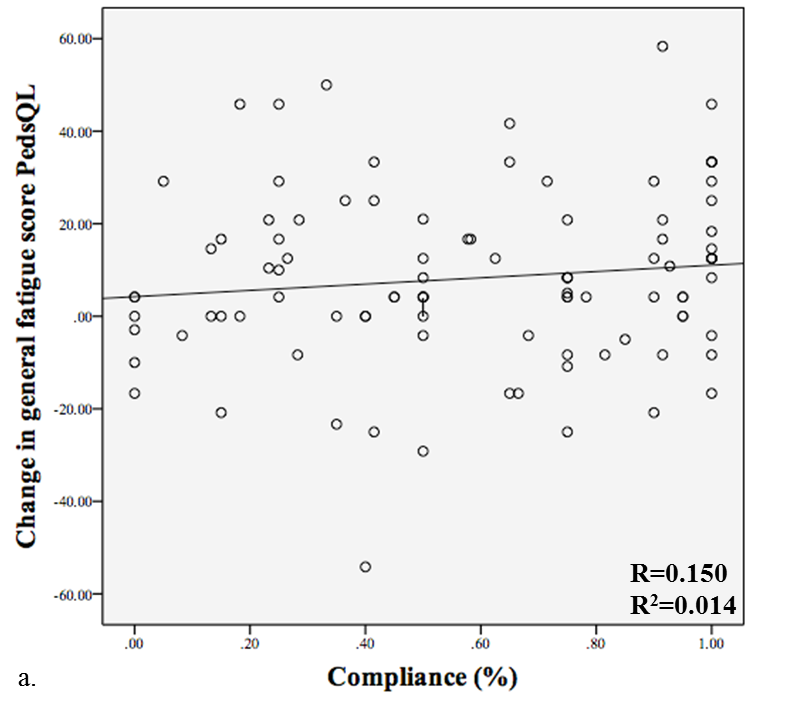

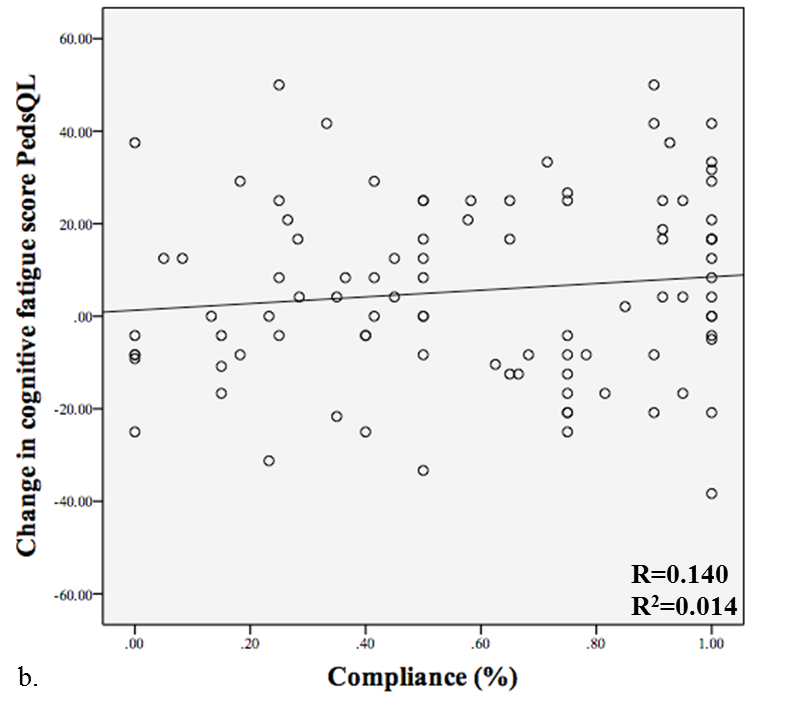


**Figure S1.** *Cont.*


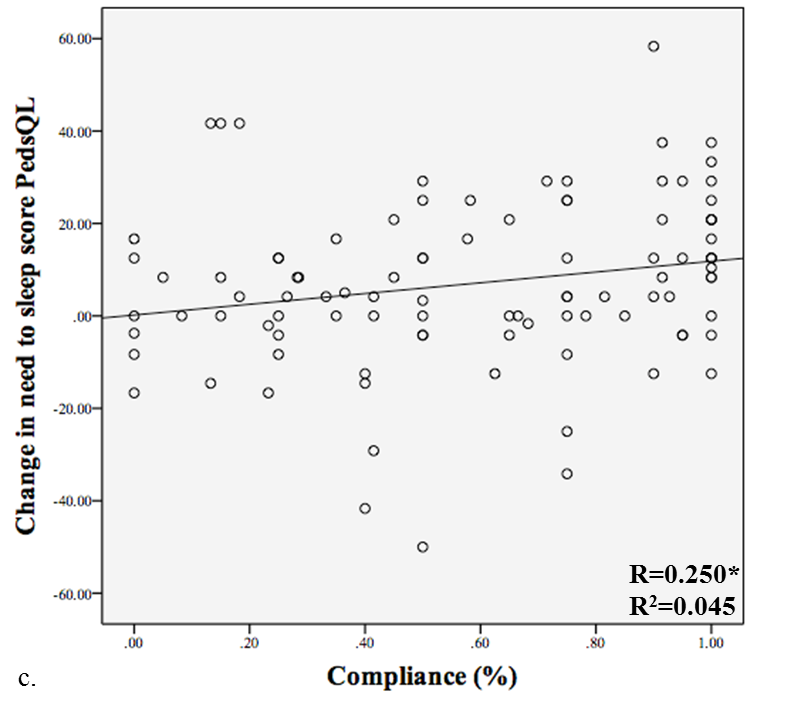


**Figure S1.** Correlation between the compliance to the advised diet and change in symptoms of fatigue (second score minus baseline score). (**a**) Correlation between compliance to the diet and change in the general fatigue score on the PedsQL; (**b**) Correlation between compliance to the diet and change in the cognitive fatigue score on the PedsQL; (**c**) Correlation between compliance to the diet and change in the need to sleep score on the PedsQL. ***** Correlation is significant at the 0.05 level (2-tailed).

© 2015 by the authors; licensee MDPI, Basel, Switzerland. This article is an open access article distributed under the terms and conditions of the Creative Commons Attribution license (http://creativecommons.org/licenses/by/4.0/).
